# Evaluation of a structured skills training group for adolescents with attention-deficit/hyperactivity disorder: a randomised controlled trial

**DOI:** 10.1007/s00787-021-01753-2

**Published:** 2021-03-15

**Authors:** Jenny Meyer, Mia Ramklint, Maria Unenge Hallerbäck, Måns Lööf, Johan Isaksson

**Affiliations:** 1 Department of Neuroscience, Child and Adolescent Psychiatry Unit, Uppsala University, Akademiska Sjukhuset, 751 85 Uppsala, Sweden; 2 School of Medical Sciences, Örebro University, Örebro, Sweden; 3 Child and Adolescent Psychiatric Clinic, Gävle, Sweden; 4 Department of Women’s and Children’s Health, Karolinska Institute Centre of Neurodevelopmental Disorders (KIND) Centre for Psychiatry Research, Karolinska Institute, Stockholm, Sweden

**Keywords:** Adolescence, ADHD, Cognitive behavioural therapy, Dialectical behavioural therapy, Psychoeducation

## Abstract

Attention-deficit/hyperactivity disorder (ADHD) in adolescence is associated with behavioural, emotional and interpersonal problems, and non-pharmacological treatments targeting these difficulties have been requested. The objective of this study was to evaluate the effectiveness and acceptance of an age-adapted structured skills training group (SSTG) for adolescents with ADHD. Adolescents (*n* = 184, ages 15–18 years) with a diagnosis of ADHD were randomly assigned to either the SSTG, which is based on dialectical behavioural therapy, or an active control group based on psychoeducation. Symptoms of ADHD, behavioural and emotional problems, functional impairment, and health-related outcomes were assessed with self-ratings and parental ratings two weeks before, two weeks after, and six months after treatment. All participants who completed the pre-treatment measurements (*n* = 164) were included in the main analyses, which were conducted using a linear mixed model. Our results demonstrated no significant group differences in favour of the SSTG for any of the study outcomes. A majority of the participants in both groups reported that they had increased their knowledge about ADHD, improved their ability to manage problems related to the diagnosis, and would recommend the treatment to others. We conclude that the SSTG seems to be acceptable for adolescents with ADHD in a clinical context. However, the treatment was not proved to be more effective or more acceptable than the psychoeducational control intervention.

**Trial registration**: http://www.isrctn.com/ISRCTN17366720,11/05/2016, retrospectively registered.

**Supplementary Information:**

The online version of this article (10.1007/s00787-021-01753-2) contains supplementary material, which is available to authorized users.

## Introduction

Attention-deficit/hyperactivity disorder (ADHD) is characterised by symptoms of inattention, hyperactivity and impulsivity, resulting in functional impairment across several life domains [1]. Specifically, adolescents with ADHD have an increased risk of academic problems, including poor schoolwork completion, school drop-out and academic failure [2, 3]. Interpersonal problems are also common among youths with ADHD [2, 4], who show an impaired ability to effectively participate in social exchange (e.g., more often express anger, have difficulties in communication and turn-taking) [5, 6]. In addition, elevated emotion reactivity, emotion regulation difficulties, and psychiatric comorbidity are common problems among adolescents with ADHD [7–9], leading to further psychosocial impairment [6, 7]. Adolescents with ADHD have low ratings on the quality of life, especially in psychosocial domains [10]. Hence, it is imperative to implement effective and acceptable treatments for adolescents with ADHD that target both ADHD symptoms and the associated problems of emotional dysregulation and social skills.

The NICE guidelines [11] recommend a stepwise care model for managing ADHD, with psychoeducation and environmental modifications regarded as first-line interventions. While pharmacological treatments have been shown to have an effect on ADHD symptoms [12], psychosocial treatments might be more beneficial for improving adaptive function in everyday life [11, 13]. Corroborating this, the NICE guidelines [11] recommend cognitive behavioural therapy (CBT), targeting social skills, problem-solving and emotion regulation, for adolescents in whom impairments persist despite medication – preferably in a group setting for cost-effectiveness. Two randomised controlled trials (RCT) have demonstrated promising results of CBT for medicated adolescents with ADHD when compared with a waiting list [14, 15], indicating that CBT treatment can reduce ADHD symptoms and functional impairment, at least in the short term. The overall effects of psychosocial treatments for adolescents with ADHD have been summarised in a systematic review by Chan and colleagues [12]. Despite some promising findings on ADHD symptoms and the improvement of skills such as planner use and homework completion, the authors concluded that the effects of psychosocial treatments on ADHD symptoms and functional impairment were inconsistent [12]. Considering the burden of emotional and relational problems among adolescents with ADHD [2–9, 16], interventions for this age group should preferably also focus on emotional dysregulation, psychiatric comorbidity and interpersonal problems [8, 17].

A CBT method which explicitly targets emotional dysregulation and relational problems is dialectical behavioural therapy (DBT), where techniques such as mindfulness, acceptance, behavioural analysis, and social skills are practiced continuously [18]. Although originally developed for patients with borderline personality disorder (BPD) [19], DBT-based methods have been proposed to be suitable for patients with ADHD due to the symptom overlap with BPD [20]. Some studies have focused specifically on mindfulness, with an aim of teaching functional skills to self-regulate attention and emotional reactivity. In a recently published meta-analysis, the findings indicated that mindfulness-based treatments could have the potential to decrease ADHD symptoms [21]. However, this meta-analysis did not include any RCTs, meaning that no firm conclusions regarding the effect of mindfulness can be drawn.

Other studies have evaluated a structured skills training group (SSTG), developed for adult patients with ADHD [22]. The SSTG combines traditional CBT with DBT and targets both the core symptoms of ADHD and associated difficulties, such as emotional dysregulation and relational problems. In uncontrolled studies, the SSTG was associated with reductions in ADHD symptoms, comorbidity and functional impairment, and improvement in personal health [22–24]. When using an RCT design with an active control group, the SSTG was found to be superior to a loosely structured discussion group regarding effects on ADHD symptoms and perceived ability to cope with deficits, but not regarding symptoms of comorbidity [25]. In the largest RCT to date, only the blinded clinical global impression ratings supported the SSTG as superior to individual clinical management [26]. These mixed findings indicate that the SSTG could be beneficial for some patients with ADHD, but also underline the impact of study design and choice of control condition. The SSTG has not yet been evaluated regarding its effectiveness for adolescents with ADHD.

To summarise, while a few studies have found promising results of CBT for adolescents with ADHD, the quality of evidence in this research area remains low [11] and there is a lack of studies evaluating group-based DBT for adolescents with ADHD. Thus, the aim of this study was to investigate the effectiveness and acceptance of an age-adapted SSTG based on DBT, for adolescents with ADHD in a clinical setting. We hypothesised that the SSTG would be superior regarding the improvement of symptoms and functioning, compared with a psychoeducational control intervention, and that the SSTG would be acceptable for adolescents with ADHD.

## Methods

### Design and procedures

This was a multi-centre RCT with two study arms comparing the SSTG with a psychoeducational control intervention performed in a clinical context. The methods have previously been described in a study protocol [27]. The recruitment, interventions and data collection were conducted at seven child and adolescent psychiatric (CAP) outpatient units in Sweden (the recruitment procedure is described in greater detail in Supplement S1). The outcome measures were assessed in self-reports and parental reports two weeks before (T1), 2 weeks after (T2) and 6 months after treatment (T3). Recruitment started in 2015 and the last follow-up measures were collected during the spring of 2019. The study was performed and reported in accordance with CONSORT guidelines (see Supplement S2 for CONSORT checklist).

### Participants

**Fig. 1:**
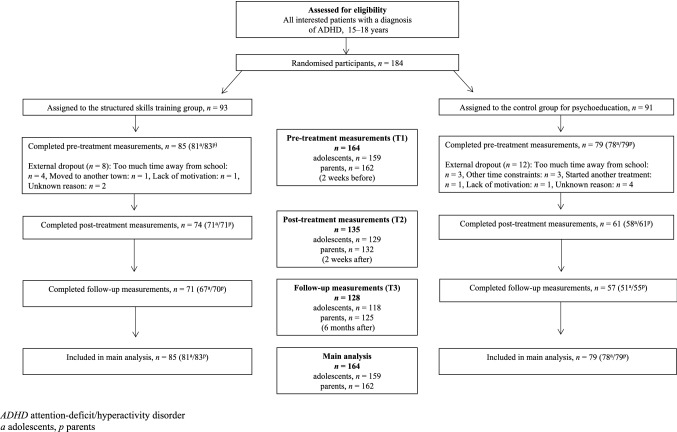
Participant flow

Sample characteristics are presented in Table 1 and the participant flow is shown in Fig. 1. The participants were patients aged 15–18 years, with a clinical diagnosis of ADHD according to the International Classification of Disease (ICD-10) [28], which was retrieved from the participants’ medical record. Assessment of study eligibility was conducted before randomisation by clinical psychologists who interviewed the adolescents and their parents at the CAP units. Exclusion criteria were severe depression, suicidality, psychosis, or bipolar disorder without stable medication, mental retardation, organic brain injury, autism spectrum disorder or current substance abuse. Any ongoing pharmacological treatment for ADHD should be stable during the treatment and the participants were requested not to take part in any other psychosocial treatment during the study period. The psychologists performed a clinical evaluation of each adolescent’s mental health status and investigated the presence of any exclusion criteria. In cases of uncertainty, the psychologist checked current comorbidities in that adolescent’s medical record.

**Table 1:** Clinical characteristics of the sample

Characteristics	SSTG (*n* = 85)	Control group (*n* = 79)
Female, *n* (%)	56 (65.8)	49 (62.0)
Mean age, years (SD)	16.46 (0.88)	16.71 (0.94)
**Clinical diagnosis of ADHD** (ICD-10), *n* (%)		
Combined	58 (68.2)	58 (73.4)
Inattentive	24 (28.2)	18 (22.8)
ADHD unspecified	3 (3.5)	3 (3.8)
**ADHD presentation** (MINI-KID), *n* (%)*,* mean no of symptoms		
Combined	33 (38.8), 15.06	40 (50.6), 15.10
Inattentive	33 (38.8), 9.42	25 (31.6), 9.40
Hyperactive-impulsive	2 (2.4), 11.00	1 (1.3), 12.00
Unspecified ADHD ^a^	17 (20.0), 5.47	13(16.5), 5.46
**Psychiatric comorbid symptoms** ^b^, *n* (%)		
Anxiety	26 (32.1)	26 (33.3)
Depression	16 (19.8)	12 (15.4)
Disruptive behaviour	33 (39.8)	24 (30.4)
At least one comorbidity	49 (62.0)	45 (57.7)
**Functional impairment** ^c^, mean (SD)		
School	7.17 (2.14)	6.89 (2.74)
Social	5.95 (2.35)	5.44 (2.58)
Home	5.17 (2.93)	4.32 (2.89)
**Medication**^d^, *n* (%)		
ADHD medications	60 (72.3)	64 (81.0)
Antidepressants & sedatives	19 (22.9)	16 (20.3)
Sleep medications	20 (24.1)	14 (17.7)
Antipsychotics & mood stabilizer	1 (1.2)	2 (2.5)

*ADHD* Attention-deficit/hyperactivity disorder, *ICD* International Classification of Disease, *MINI-KID* Mini International Neuropsychiatric Interview for Children and Adolescents, *SD* standard deviation, *SSTG* structured skills training group

^a^Unspecified ADHD includes participants who did not fulfill the criteria for any of the main presentations in the MINI-KID interview

^b^Affective comorbidity was assessed using self-ratings (n = 159) on the subscales in the Hospital Anxiety and Depression scale. A score of ≥ 10 points on the subscale of depression was classified as depression and a score of ≥ 12 points on the subscale of anxiety was classified as anxiety. Disruptive behaviour was identified based on parental ratings (n = 162) on the subscale of conduct problems in the Strengths & Difficulties Questionnaire, where a score of ≥ 4 was classified as disruptive behaviour. At least one comorbidity was based on the number of participants for whom both self-ratings and parental ratings were available (SSTG: n = 79; control group: n = 78)

^c^ Functional impairment was assessed using parental ratings on the Child Sheehan Disability Scale, where each area ranges from 0 to 10. A score of 1–3 = little impairment, 4–6 = moderate impairment and ≥ 7 = high impairment

^d^ Parental reports of ADHD medication, including methylphenidate, atomoxetine, lisdexamfetamine or guanfacine. Parental reports of other psychopharmacological medication included *antidepressants*: fluoxetin, sertralin, escitalopram, bupropion; *sedatives*: prometazin, alimemazin, hydroxizin; *sleep medications*: melatonine, propiomazin; *mood stabilizers*: lithium; *antipsychotics*: olanzapine, aripiprazole, risperidone

Since the adolescents were diagnosed with ADHD prior to the study (in some cases, several years earlier), each participant’s current presentation of ADHD symptoms was assessed by clinical psychologists at the CAP units using the section for ADHD in the Mini International Neuropsychiatric Interview for Children and Adolescents (MINI-KID) [29]. The current presentation of symptoms was based on the number of prevalent symptoms in the preceding 6 months and assessed in accordance with the fifth edition of the Diagnostic and Statistical Manual of Mental Disorders (DSM-5) [1]. Participants who fulfilled fewer than six symptoms (< five symptoms for adolescents aged 17 years and older) of both inattention and hyperactivity/impulsivity, were categorised as unspecified ADHD. As shown in Table 1, the participants’ current presentations of symptoms revealed a transition in symptomatology, where the predominantly inattentive presentation and unspecified ADHD were more prevalent, according to the MINI-KID assessment, compared with the prior clinical diagnoses. A majority of the participants (76.5%) had pharmacological treatment for ADHD when entering the study and more than one-third (35.8%) had additional psychopharmacological treatment. Overall, the participants had moderate to high levels of functional impairment, and about two-thirds (59.9%) of the sample reported other psychiatric symptoms that indicated psychiatric comorbidity.

In all, 184 participants were randomised, and 164 participants completed the T1 measurements. Randomised participants who did not complete T1 were defined as external dropouts and not included in the statistical analyses. Those who completed T1 but did not complete any of the assessments post-treatment were defined as internal dropouts and included in the analyses.

### Interventions

#### SSTG

The treatment is an age-adapted version of a manualised DBT-based group programme originally developed for adults with ADHD [30, 31] and consists of 14 weekly 2-h sessions, where each session has a specific theme. For more detailed information about the SSTG, see Supplement S3. SSTG includes elements of psychoeducation and strategies for managing difficulties related to ADHD. DBT elements, such as mindfulness, acceptance, behavioural analysis and social skills, are incorporated and practiced during treatment. Age adaptions, such as simplified language, more practical exercises, and less theory, were made prior to treatment initiation [27]. Each group was led by two therapists, who were clinicians working at the CAP units, of which at least one was a psychologist and one was trained in DBT. Minor modifications of the age-adapted SSTG were made after the first treatment period (*n* = 19), but as no significant outcome differences were found between the groups pre- and post-modification, all SSTG participants were merged into one group. The group leaders registered the attendance and completed homework assignments of each participant (max = 13 assignments).

##### Psychoeducational control intervention

The control group followed a manual-based psychoeducational group programme (SKILLS), designed by JI and ML. The intervention consists of three 2-h sessions, where the main focus is psychoeducation about ADHD, including information about ADHD symptomatology, strengths and challenges with ADHD, sleep and diet, stress management, problem-solving, and structuring daily life routines. For more detailed information about the psychoeducational intervention, see Supplement S3. The participants also received a book describing tools to facilitate schoolwork. Each group was led by two therapists, who were clinicians working at the CAP units. DBT-related components were not included in the control intervention. The group leaders registered the attendance and completed homework assignments of each participant (max = 2 assignments).

##### Treatment fidelity

All therapists (psychologists, psychiatrist, psychiatric nurses and social workers) were trained in the respective method before delivering the intervention. The training included education covering the manual and practical exercises. The manual was also discussed at yearly meetings with the therapists involved in the project. Continuous supervision for each method was offered during the study period, whereby the therapists could receive support and guidance for upcoming sessions, to stay adherent to the method. The training and supervision of the SSTG were provided by a clinical psychologist and psychotherapist, educated in DBT and with experience of the SSTG. Adherence to the SSTG method was assessed by ratings of 27 video-recorded sessions. Two clinical psychologists with expertise in CBT and the SSTG method performed adherence ratings on a five-point scale, ranging from 1 (unacceptable) to 5 (excellent). The average adherence was considered acceptable to good (*M* = 3.57, *SD* = 0.34); for information about the adherence ratings, see Supplement S4. The training in and supervision of the psychoeducational control intervention was provided by a clinical nurse and psychotherapist, with expertise in the method. The group leaders were carefully informed to stick strictly to the manual and use the PowerPoint presentation created for each respective session. No formal adherence assessment was performed for the therapists in the control group.

### Measurements

#### Current medication

Current ADHD medication was reported by parents at T1, T2 and T3. For this study, reported ADHD medication was categorised as: stable medication (i.e., continued with ADHD medication throughout the study period), major change of medication (i.e., either stopped or started using ADHD medication) or no medication (i.e., did not use any ADHD medication).

### Primary outcomes

ADHD symptoms were assessed using self-ratings and parental ratings on the *Adult ADHD Self-Report Scale for Adolescents (ASRS-A)* [32, 33]. The questionnaire contains 18 items corresponding to the diagnostic criteria of ADHD, measured on a 5-point scale from 0 (never) to 4 (very often), with higher scores indicating more symptoms. The ASRS-A has shown promising psychometric properties in clinical populations [32, 33]. In this study, Cronbach’s alpha (*α*) indicated good to excellent internal consistency (*α* = 0.91 for self-ratings and *α* = 0.89 for parental ratings).

Functional impairment was assessed using self-ratings and parental ratings on the *Child Sheehan Disability Scale (CSDS)* [34]. The self-rating scale assesses functional impairment in three areas (school, social activities, and home). The parental rating scale assesses the impact of the adolescents’ symptoms in five areas (school, social activities, home, parents’ work, and parents’ social activities). The impairment in each area is measured on an 11-point scale from 0 (not at all) to 10 (very much), with higher scores indicating more impairment. The CSDS has shown good validity in a sample of children and adolescents with psychiatric disorders [34] and displayed acceptable to good internal consistency (*α* = 0.77 for self-ratings and *α* = 0.83 for parental ratings) in this study.

The impact of ADHD-related symptoms on each adolescent’s wellbeing was measured using the questionnaire *Impact of ADHD Symptoms (IAS)*, constructed for this study. IAS is a six-item self-rating scale where the adolescent indicates the extent to which their wellbeing during the preceding week has been affected by impulsive behaviours, difficulties starting and completing assignments on time, hyperactivity, sleep problems, emotional dysregulation and stress. Each item is answered on an 11-point scale from 0 (not at all) to 10 (very much), with higher scores indicating a greater impact of symptoms. IAS displayed good internal consistency (*α* = 0.80) in this study.

Quality of life was measured using the *Global Quality of Life scale (GQL)* [35]. The adolescents answered the question “How is your life right now?” on an 11-point scale from 0 (the worst imaginable life situation) to 10 (the best imaginable life situation). The GQL has shown acceptable psychometric properties in a clinical sample of adults with psychiatric disorders [35].

Mindfulness was measured using the *Five Facet Mindfulness Questionnaire (FFMQ)* [36, 37], with 29 items measured on a 5-point self-rating scale from 1 (never/almost never) to 5 (always), where higher scores reflect a higher level of mindfulness. The construct validity of the scale is supported [36, 37] and the internal consistency for the total scale was acceptable (*α* = 0.77) in this study.

### Secondary outcomes

Total difficulties of behavioural and emotional problems were assessed using self-ratings and parental ratings, based on the total difficulty score from the *Strengths & Difficulties Questionnaire (SDQ)* [38]. The score encompasses 20 statements about the occurrence of ADHD symptoms and emotional, conduct and peer problems, measured on a 3-point scale from 0 (not true) to 2 (certainly true), where higher scores indicate more severe problems. The Swedish version of the SDQ has shown adequate validity [39] and an acceptable internal consistency was observed in this study (*α* = 0.78 for self-ratings and *α* = 0.72 for parental ratings). In the descriptive statistics, the parent-rated subscale of conduct problems was used to identify patients with elevated disruptive behaviours, using the cut-off value suggested by previous research [40].

Symptoms of anxiety and depression were measured using self-ratings on the *Hospital Anxiety and Depression Scale (HADS)* [41]. The questionnaire contains 14 statements measured on a 4-point scale, ranging from 0 to 3, where higher scores indicate a greater occurrence of symptoms. The HADS has shown good psychometric properties in a sample of adolescents [42] and the internal consistency was good in this study (*α* = 0.85). In the descriptive statistics, the subscales of depression and anxiety were used to identify patients reporting clinical levels of symptoms of these respective conditions, using the cut-off values suggested by previous research [42].

Perceived stress was measured using self-ratings on the *Pressure Activation Stress* scale *(PAS)* [43]. The questionnaire consists of 11 items, measuring stress symptoms on a 5-point scale from 0 (never) to 4 (always), where higher scores indicate more stress. The scale has displayed a promising face validity and good internal consistency [43], which was confirmed in this study (*α* = 0.88).

Sleep problems were measured using self-ratings on the *Karolinska Sleep Questionnaire (KSQ)* [44]. The KSQ includes seven statements about difficulties falling asleep and waking up. Each item is measured on a 6-point scale from 0 (never) to 5 (always), where higher scores indicate more sleep problems. The KSQ has shown adequate psychometric properties [44] and the internal consistency was good in this study (*α* = 0.83).

### Treatment acceptability

After the final group session, the participants answered a questionnaire about how they had perceived and responded to the group intervention. The questionnaire was inspired by a questionnaire from the Swedish SSTG manual for adults [31], which was adapted and shortened by the research team to be used for adolescents, and included four questions about if their knowledge about ADHD had increased, if they were more able to manage problems related to ADHD, how much they had benefitted from the treatment, and if they would recommend it to others.

### Randomisation

Participants were randomised to one of the two treatment conditions using a computer-generated allocation sequence (https://www.randomizer.org) with separate sequence lists for each treatment centre, where the participants were randomly assigned at a 1:1 ratio. Codes were used to ensure information confidentiality and participant anonymity and the principal investigator performed treatment allocation based on the codes. Participants were not blinded to the treatment condition.

### Statistical analyses

The appropriate sample size was determined based on a power calculation to detect a group difference of at least seven points on the ASRS-A and a difference of at least four points on the CSDS. Specifically, to obtain a moderate effect size (d ≥ 0.50) with a power of 80%, using a two-tailed t-test, *α* = 0.05, the final sample size had to be at least 100 participants (a detailed description of the power calculation is provided in the study protocol) [27]. All analyses were conducted with the Statistical Package for the Social Sciences (IBM SPSS), version 26. Normal distribution was explored for each group separately, and after adjusting for single outliers by changing the outlier to the nearest value (*n* = 1 on two scales and *n* = 2 on one scale), all the scales were regarded as normally distributed, i.e., the values of *Z*_Skewness_ and *Z*_Kurtosis_ were in the range of -1.96–1.96 [45]. Descriptive statistics were used to describe the sample. Attrition analyses and differences between the two groups regarding baseline variables, medication, attendance and homework completion were assessed using independent-samples t-tests and chi-squared tests. Treatment outcomes were compared between the different treatment sites, using one-way analysis of variance (ANOVA) with Tukeys’ HSD as a post hoc test.

The main analysis from the published study protocol [27] was changed from a general linear model to a linear mixed model because of the mixed model’s advantages of using all available data from participants and enabling inclusion of random effects [46]. Multiple imputation of missing data was not used. In the linear mixed model, a major change in medication (i.e., stopped or started ADHD medication) was used as a covariate, the participants’ baseline values as random intercept, time and treatments as fixed factors, and time by treatment as an interaction term. The effectiveness of the treatment was estimated by contrasting the longitudinal mean changes between the SSTG and the control group at each timepoint. Specifically, the change in symptoms from T1 to T2 and the change from T1 to T3 were compared separately. Within-group differences were explored by calculating the mean changes within each group between each timepoint. A sensitivity analysis was conducted on the completers, i.e., those who attended at least two-thirds of the sessions (SSTG, *n* = 54; control group, *n* = 62). In addition, a sensitivity analysis was conducted for the IAS item regarding the impact of emotional dysregulation, to investigate group differences and within-group changes in this specific outcome. Cohen’s d was used as a measure of effect size, using the observed values for each group. Treatment acceptability was investigated using descriptive statistics for each group. As a supplementary analysis, group differences regarding acceptability were explored using the chi-squared test. All the reported results were considered significant at the 5% level.

## Results

### Sample characteristics

No significant differences were found between the two groups at baseline. Moreover, the attrition analysis showed no differences in the baseline values between the internal dropouts and those who completed post-measurements. No group differences were found in regard to patterns/changes in ADHD medication during the study. More specifically, a majority in both groups had a stable medication during the study (62% in the SSTG and 72% in the control group), nearly one fifth underwent a major change in medication (21% in the SSTG and 18% in the control group) and somewhat fewer had no ADHD medication throughout the study (17% in the SSTG and 10% in the control group). The mean attendance in the SSTG was 8.7 sessions (62% of all included sessions), while the mean attendance in the control group was 2.3 sessions (76% of all included sessions), revealing a significant group difference in the proportion of attendance (*t* = 2.76*, p* = 0.007). Although a substantial proportion of data was missing regarding homework completion, the available data (n = 93) indicated no group difference. More specifically, 45% of the homework assignment were completed in the SSTG, while 43% of the homework assignments were completed in the control group. No differences in the treatment outcomes were found between the treatment sites.

### Primary outcomes

Between- and within-group differences are presented in Table 2. No differences were observed between the two groups regarding changes in the primary outcomes (effect sizes indicated no to small effects, *d* = 0.01 to 0.33). Within-group changes were found in the SSTG, with reductions of ADHD symptoms and functional impairment in both self-reports (T1-T3) and parental reports (T1-T2 and T1-T3). These results were confirmed in the sensitivity analysis of completers. For the control group, only parents reported reductions of ADHD symptoms and functional impairment (T1-T3), and the sensitivity analysis of completers showed a reduction of parentally rated ADHD symptoms at T2 as well. Cohen’s d indicated moderate effects for the decrease of ADHD symptoms in the SSTG according to parental reports (*d* = 0.59 [T1–T2], *d* = 0.62 [T1–T3]) and small effects for the other significant within-group differences (*d* = 0.26–0.45). The sensitivity analysis regarding the impact of emotional dysregulation revealed neither group differences nor within-group changes.

**Table 2:** Primary outcomes with Estimated Marginal Means (EMM) and 95% Confidence Interval (CI) obtained from the linear mixed model; adjusted for changes in medication

Variable	Baseline (T1) *EMM (CI)*	Post (T2) *EMM (CI)*	Follow-up (T3) *EMM (CI)*	T1-T2; *B (CI)*	T1–T3; *B (CI)*	Time by group interaction T1–T2; *B (CI)* T1–T3; *B (CI)*
ADHD-symptoms (adolescent)						
SSTG	40.05 (36.57; 43.52)	38.97 (35.47; 42.47)	36.89 (33.37; 40.41)	1.08 (− 1.08; 3.23)	3.16 (0.97; 5.35)*	− 0.95 (− 4.13; 2.23)
Control group	43.72 (39.90; 47.55)	43.60 (39.76; 47.44)	43.59 (39.66; 47.52)	0.13 (− 2.21; 2.46)	0.14 (− 2.35; 2.63)	− 3.02 (− 6.34; 0.30)
ADHD-symptoms (parent)						
SSTG	44.37 (41.44; 47.31)	39.77 (36.84; 42.71)	38.03 (35.08; 40.98)	4.60 (2.61; 6.58)***	6.34 (4.33; 8.36)***	− 2.73 (− 5.65; 0.19)
Control group	41.79 (38.58; 45.00)	39.92 (36.71; 43.13)	37.94 (34.67; 41.21)	1.87 (− 0.27; 4.01)	3.85 (1.61; 6.09)***	− 2.50 (− 5.51; 0.52)
Functional impairment (adolescent)						
SSTG	13.85 (12.03; 15.67)	12.34 (10.50; 14.19)	11.30 (9.44; 13.16)	1.51 (− 0.07; 3.09)	2.55 (0.95; 4.16)**	− 1.09 (− 3.42; 1.24)
Control group	14.64 (12.65; 16.64)	14.22 (12.21; 16.24)	13.37 (11.27; 15.47)	0.42 (− 1.29; 2.13)	1.28 (− 0.54; 3.10)	− 1.28 (− 3.70; 1.15)
Functional impairment (parent)						
SSTG	26.52 (23.67; 29.36)	22.94 (20.09; 25.78)	23.56 (20.70; 26.43)	3.58 (1.65; 5.51)***	2.95 (0.99;4.91)**	− 2.07 (− 4.91; 0.77)
Control group	24.88 (21.77; 28.00)	23.37 (20.26; 26.48)	21.39 (18.21; 24.56)	1.51 (− 0.57; 3.59)	3.49 (1.31; 5.66)**	0.54 (− 2.39; 3.47)
Impact of ADHD-symptoms						
SSTG	29.61 (26.21; 33.02)	27.60 (24.15; 31.04)	27.57 (24.10. 31.04)	2.02 (− 0.62; 4.65)	2.04 (− 0.64; 4.72)	− 2.05 (− 5.92; 1.83)
Control group	30.87 (27.13; 34.61)	30.90 (27.15; 34.65)	30.59 (26.69; 34.48)	− 0.03 (− 2.87; 2.81)	0.28 (− 2.76; 3.32)	− 1.76 (− 5.81; 2.29)
Quality of life						
SSTG	5.94 (5.38; 6.51)	6.01 (5.44; 6.59)	6.05 (5.46; 6.63)	− 0.07 (− 0.61; 0.47)	− 0.10 (− 0.65; 0.45)	− 0.09 (− 0.89; 0.70)
Control group	5.82 (5.20; 6.44)	5.99 (5.36; 6.61)	6.13 (5.47; 6.79)	− 0.17 (− 0.75; 0.42)	− 0.31 (− 0.94; 0.31)	− 0.20 (− 1.04; 0.62)
Mindfulness						
SSTG	85.57 (82.52; 88.61)	85.36 (82.28; 88.44)	84.21 (81.11; 87.31)	0.21 (− 2.05; 2.47)	1.36 (− 0.93; 3.65)	− 0.86 (− 4.20; 2.48)
Control group	80.62 (77.27; 83.97)	81.27 (77.89; 84.65)	80.57 (77.10; 84.05)	− 0.65 (− 3.12; 1.81)	0.05 (− 2.56; 2.65)	− 3.31 (− 4.78; 2.15)

*ADHD* Attention-deficit/hyperactivity disorder, *SSTG* structured skills training group; * *p* < 0.05; ** *p* < 0.01; *** *p* < 0.001

### Secondary outcomes

Between- and within-group differences are presented in Table 3. No differences were observed between the two groups regarding changes in total difficulties, symptoms of depression and anxiety, or perceived stress (effect sizes indicated no to small effects, *d* = 0.09 to 0.36). One between-group difference was found regarding sleep problems, in favour of the control group. This difference was only observable at T2 and was neither preserved at T3 nor confirmed in the sensitivity analysis of completers. No within-group differences in sleep problems were found in either of the groups. Regarding self-reported symptoms of anxiety and depression, a within-group difference was found for the SSTG (T1-T3). In the sensitivity analysis, this was attenuated to a trend (*B* = 1.42, *p* = 0.064). Within-group differences of total difficulties were observed for both groups, indicating a decrease of emotional and behavioural difficulties. These results were preserved in the sensitivity analysis. The decrease of total difficulties reported by parents in the SSTG indicated moderate effects (*d* = 0.68 [T1–T2], *d* = 0.69 [T1–T3]), while the other significant within-group differences indicated small effects (*d* = 0.25–0.46).

**Table 3:** Secondary outcomes with Estimated Marginal Means (EMM) and 95% Confidence Interval (CI) obtained from the linear mixed model; adjusted for changes in medication

Variable	Baseline (T1) *EMM (CI)*	Post (T2) *EMM (CI)*	Follow-up (T3) *EMM (CI)*	T1–T2 *B (CI)*	T1–T3 *B (CI)*	Time by group interaction T1–T2; *B (CI)* T1–T3; *B (CI)*
Total difficulties (adolescent)						
SSTG	16.54 (15.10; 17.99)	15.87 (14.41; 17.33)	14.96 (13.49; 16.43)	0.67 (− 0.39; 1.72)	1.58 (0.51; 2.65)**	− 0.81 (− 2.36; 0.75)
Control group	18.17 (16.58; 19.76)	18.31 (16.71; 19.91)	16.07 (14.42; 17.72)	− 0.14 (− 1.28; 1.00)	2.10 (0.89; 3.31)***	0.52. (− 1.10; 2.14)
Total difficulties (parent)						
SSTG	17.71 (16.35; 19.07)	14.81 (13.45; 16.17)	14.30 (12.93; 15.67)	2.90 (1.77; 4.02)***	3.41 (2.27; 4.55)***	− 0.97 (− 2.63; 0.68)
Control group	16.85 (15.36; 18.33)	14.92 (13.44; 16.41)	14.33 (12.80; 15.85)	1.93 (0.71; 3.14)**	2.52 (1.26; 3.79)***	− 0.89 (− 2.59; 0.82)
Symptoms of depression & anxiety						
SSTG	15.29 (13.45; 17.12)	14.74 (12.89; 16.59)	13.67 (11.81; 15.54)	0.55 (− 0.76; 1.86)	1.61 (0.28; 2.94)*	− 0.36 (− 2.29; 1.57)
Control group	16.61 (14.59; 18.62)	16.42 (14.39; 18.44)	15.99 (13.90; 18.08)	0.19 (− 1.23; 1.61)	0.62 (− 0.89; 2.13)	− 1.00 (− 3.01; 1.02)
Sleep problems						
SSTG	16.98 (15.07; 18.88)	18.08 (16.15; 20.00)	17.92 (15.98; 19.87)	− 1.10 (− 2.59; 0.38)	− 0.95 (− 2.46; 0.55)	2.34 (0.15; 4.53)*
Control group	16.66 (14.56; 18.75)	15.42 (13.31; 17.53)	16.37 (14.19; 18.56)	1.24 (-0.37; 2.85)	0.28 (− 1.43; 1.99)	1.24 (− 1.04; 3.52)
Perceived stress						
SSTG	22.54 (20.36; 24.72)	22.93 (20.73; 25.14)	22.14 (19.92; 24.36)	− 0.39 (− 2.07; 1.29)	0.40 (− 1.13; 2.11)	− 0.26 (− 2.74; 2.22)
Control group	24.95 (22.56; 27.35)	25.60 (23.19; 28.01)	24.15 (21.66; 26.65)	− 0.65 (− 2.48; 1.17)	0.80 (− 1.14; 2.74)	0.40 (− 2.19; 2.99)

*SSTG* structured skills training group; * *p* < 0.05; ** *p* < 0.01; *** *p* < 0.001

### Treatment acceptability

Treatment acceptability is presented in Table 4. A majority of the responding adolescents in both groups reported increased knowledge about ADHD and increased ability to manage ADHD-related problems. Most participants reported having benefited from the group intervention and would recommend it to others. The supplementary analysis revealed no significant group differences regarding acceptability.

**Table 4:** Acceptance ratings

Item					
My knowledge about ADHD has increased ^a^	Not true	Somewhat true	Certainly true		
SSTG, *n* = 55	7.3%	60.0%	32.7%		
Control group, *n* = 55	7.3%	60.0%	32.7%		
I am more able to manage problems related to ADHD ^a^	Not true	Somewhat true	Certainly true		
SSTG, *n* = 55	10.9%	69.1%	20.0%		
Control group, *n* = 50	16.0%	74.0%	10.0%		
Would you recommend others to participate in this group?	Yes	No			
SSTG, *n* = 58	87.9%	12.1%			
Control group, *n* = 59	89.8%	10.2%			
How much of a benefit do you think the treatment had for you?	0 = no benefit	1	2	3	4 = very great benefit
SSTG, *n* = 59	5.1%	6.8%	25.4%	44.1%	18.6%
Control group, *n* = 61	6.5%	11.5%	27.9%	42.6%	11.5%

*ADHD* Attention-deficit/hyperactivity disorder, *SSTG* structured skills training group

^a^ The answer “I don’t know” (*n* = 5 for SSTG and *n* = 6 and *n* = 9 for the control group in the respective cases) was regarded as missing data and excluded from the analysis

## Discussion

This is the first RCT evaluating the effectiveness of a DBT-based structured skills training group for adolescents with ADHD, using an active control group based on psychoeducation. No group differences in favour of the SSTG were observed in any of the study outcomes. A majority of the participants in both groups reported that they had increased their knowledge about ADHD and improved their ability to manage problems related to the diagnosis.

Our hypothesis of the SSTG being superior to the psychoeducational control intervention was not supported. Indeed, the only significant group difference that was observed was in regard to sleep problems and was in favour of the control group. However, the absence of a within-group difference in both groups indicates that neither of the interventions had any effect on sleep problems. The lack of group differences in our study is partly in line with the results from previous studies using an active control group [25, 26, 47] and the small effect sizes found in our study corroborate previous literature comparing active interventions [48]. Previously reported group differences have been more prominent in RCTs using non-active control groups, such as a waiting list [14, 15]. The use of an active control group may decrease the risk of confounders from common factors, e.g., attention from a therapist and being in a group, and enables the drawing of conclusions regarding the effect of one treatment relative to another. However, the design makes it difficult to draw conclusions about the absolute effects of each intervention [49], and the lack of group differences in this study has several possible explanations that need to be discussed.

First, the relatively low attendance and homework completion levels in the SSTG indicate that many of the participants did not receive the entire treatment programme. Even though the analyses of completers confirmed the lack of group differences, we cannot rule out that we would have yield other results if the majority of participants had attended all sessions and performed the homework as intended. The group format prevents the participants from catching up on missed sessions and has somewhat limited flexibility for individual adaption. The addition of individual sessions in parallel with the group sessions [18] could enable both compensation for missed content and create more room for tailoring the practice for individual needs [50]. Second, the SSTG includes several themes, with new concepts and skills introduced over a relatively brief period of time. Accordingly, more extensive practice, including recurrent performance feedback on each skill, might be needed to enable behavioural changes [51]. Moreover, the involvement of parents and teachers in the treatment could be warranted, to support the adolescents’ practice and use of the skills in their everyday life [50]. For example, the addition of parental training could strengthen communication and collaboration to improve self-management [12]. Third, motivational interviewing has been included in CBT for adolescents with ADHD [14, 15] and might increase their motivation and adherence to the treatment [12]. Fourth, the study population was heterogenous in regard to symptom burden, comorbidity and functional impairment. Considering the focus of the SSTG, it is possible that a more homogenous study population, e.g., only patients with pronounced problems of emotional dysregulation and interpersonal problems, would have yielded other results.

Both the SSTG and the psychoeducational control intervention were perceived as beneficial by a majority of the participants, who reported increased knowledge about ADHD and an improved ability to manage difficulties related to the diagnosis. In addition, both groups demonstrated significant decreases of ADHD symptoms, functional impairment, and behavioural and emotional problems, which were preserved six months after the treatment. Although the within-group changes could be a result of maturation or regression to the mean, we cannot rule out that both interventions had some effect. Possibly, psychoeducation (which was included in both groups) may have produced more benefits than anticipated when designing the study. Psychoeducation has recently been proposed to result in symptom relief for patients with ADHD [52].

This study has some limitations that need to be addressed. Since the randomisation was performed before the pre-treatment measures there was some dropout between randomisation and T1. This could have introduced a bias of selective drop-out and increased the risk of systematic differences between the groups. However, no group differences were found in the study variables at T1. Moreover, we had a relatively large dropout, which is partly in line with previous studies and may reflect the nature of the disorder [26, 53]. Since the main analysis and the sensitivity analysis of completers showed similar results, our findings appear to be valid and not confounded by internal dropouts. Furthermore, the psychoeducational control intervention had fewer sessions than the SSTG, making time a possible confounding factor in this study. However, since a few sessions of psychoeducation are recommended as an intervention for youths with ADHD [11], this was considered an appropriate control condition. The use of a non-active control group would have enabled us to draw firmer conclusions about absolute treatment effects; at the same time, from a clinical perspective, it was considered advantageous that all participants were offered an intervention, especially given the longitudinal design. Moreover, the sessions of the psychoeducational control intervention were not recorded and the risk of some treatment contamination cannot be completely ruled out. However, the procedure included several steps to strengthen treatment fidelity, including continuous education in the method, supervision and guidance, information to stick strictly to the manual, and the use of two therapists in each group. In addition, the number of group sessions delivered was registered for all groups, confirming that the participants did not receive more sessions than were included in the manual.

Since neither parents nor adolescents were blinded to the intervention, their expectations of each intervention may have biased their ratings. The addition of a more objective evaluation of the outcomes, e.g., by a clinician blinded to the treatment conditions, could have strengthened the conclusions. Considering the focus of the SSTG, the inclusion of a validated measure that more directly assesses emotional dysregulation would also have been warranted. Lastly, the characteristics of the sample might limit the generalisability of the findings in this study. A majority of this sample had an ongoing pharmacological treatment for ADHD, limiting the generalisability to non-medicated patients. Further, this sample included patients who were categorised as unspecified ADHD (i.e., did not fulfil criteria for any of the main presentations), and it is possible that there was limited room for further improvement for these adolescents. Despite this, the ADHD symptom ratings among our participants largely corresponded to the values shown in previous validation studies of the ASRS-A in a clinical population of Swedish adolescents with ADHD [32, 33]. Two thirds of the sample reported symptoms of psychiatric comorbidity, and more than one third had psychopharmacological treatment other than ADHD medication. Although there were no group differences regarding these factors at baseline, potential changes of psychopharmacological treatment other than ADHD medication could have influenced the outcomes. Hence, it is a limitation that this factor was not included in the analyses. Moreover, a majority of the participants were girls (64%), which does not correspond to the gender distribution of ADHD [1]. This might reflect a self-selection bias; a similar gender distribution has also been reported in previous evaluations of the SSTG for adults with ADHD [24, 25]. However, previous research on psychosocial treatment for childhood ADHD have shown that gender does not seem to have a decisive role in treatment outcomes [14, 54]. Although we know that young individuals with ADHD in Sweden are more likely to have separated parents with lower education [55] and that the CAP units involved in this study have large uptake areas with patients from both rural and urban settings, the lack of information about socioeconomic status and treatment history for this particular sample was a limitation.

The main strength of the study was the RCT design, which decreased the risk for systematic differences and confounders. The multi-centre study within a clinical context increased ecological validity, and the longitudinal design enabled us to evaluate the persistence of the results. The use of both self-reports and parental reports contributed to a broader perspective on the outcomes. Results for all outcome measures have been presented in this paper, to avoid reporting bias [56].

In conclusion, the SSTG was perceived as helpful and seems to be acceptable for adolescents with ADHD in a clinical context. However, the treatment was not proved to be more effective or more acceptable than the psychoeducational control intervention. Though our findings indicate that the SSTG should not be recommended as part of the standard care for all adolescents with ADHD, more research is needed to explore if this treatment might be more beneficial for certain subgroups among patients with ADHD. In addition, further age adaptions of the SSTG, such as involvement of parents and more extensive practice, should be considered.

## Supplementary Information

Below is the link to the electronic supplementary material.

Electronic supplementary material 1 (DOCX 20 kb)

Electronic supplementary material 2 (DOC 220 kb)

Electronic supplementary material 3 (DOCX 21 kb)

Electronic supplementary material 4 (DOCX 14 kb)

## Data Availability

Consent for data sharing outside the research team was not obtained. However, reasonable requests for patient-level data should be made to the corresponding author (JI) and will be considered after discussion with the ethical board. Relevant data is included in the manuscript.
