# Supplementary material for: Evaluation of a structured skills training group for adolescents with attention-deficit/hyperactivity disorder: a randomised controlled trial
Source: Eur Child Adolesc Psychiatry. 2021 Mar 15;31(7):1–13. doi: 10.1007/s00787-021-01753-2 (PMC9343260; doi:10.1007/s00787-021-01753-2)
Supplement: Supplementary file 1 — Electronic supplementary material 1 (DOCX 20 kb) [file 787_2021_1753_MOESM1_ESM.docx]

**Supplement S1** Recruitment

The recruitment was conducted at child and adolescent psychiatry (CAP) outpatient units in seven medium-sized cities in Sweden. The CAP units involved in this project have large uptake areas, with patients from both rural and urban settings.

Recruitment was conducted using written information about the study in waiting rooms, as well as verbal and written information delivered by the clinical staff. The information stated that the study aims were to investigate whether psychological group treatment could lead to reduced symptom burden and improved health, to increase insights into and understanding about ADHD, and to teach strategies for coping with difficulties that are common among adolescents with ADHD. Further, the patients were informed about the randomisation and given a short summary of the content and length of each treatment. It was also stated that the structured skills training group was going to be video-recorded in order to review the therapists. The patients were informed that participation in the study included answering questionnaires at repeated occasions, and that information about their clinical diagnoses would be obtained from their medical records. In addition, the patients were informed that participation in the study was voluntary and that they had the right to end participation at any time. Information about data storage and confidentiality was also included.

All interested patients between 15 and 18 years who had an ADHD diagnosis were invited to a meeting together with their parents at their local CAP unit. In this meeting clinical psychologists at the CAP units assessed eligibility. The participants had the opportunity to ask questions and the study information was repeated and explained further. The psychologists performed a clinical evaluation of the adolescent’s mental health status and investigated the occurrence of any exclusion criteria. In cases of uncertainty regarding possible exclusion criteria, the psychologist checked current comorbidities in the adolescent’s medical record. Exclusion criteria were severe depression, suicidality, psychosis, bipolar disorder without stable medication, mental retardation, organic brain injury, autism spectrum disorder, or current substance abuse. Any ongoing pharmacological treatment for ADHD had to be stable during the study period and the participants were requested not to take part in any other psychological treatment during that period.

The clinical psychologists also assessed current presentation of ADHD symptoms using the section for ADHD in Mini International Neuropsychiatric Interview for Children and Adolescents (MINI-KID). The current presentation of symptoms was based on the number of prevalent symptoms in the preceding six months, using the DSM-5 criteria. If the participants were eligible for the study and wanted to participate, written informed consent was obtained.
