# Supplementary material for: Evaluation of a structured skills training group for adolescents with attention-deficit/hyperactivity disorder: a randomised controlled trial
Source: Eur Child Adolesc Psychiatry. 2021 Mar 15;31(7):1–13. doi: 10.1007/s00787-021-01753-2 (PMC9343260; doi:10.1007/s00787-021-01753-2)
Supplement: Supplementary file 3 — Electronic supplementary material 3 (DOCX 21 kb) [file 787_2021_1753_MOESM3_ESM.docx]

**Supplement S3** Themes and content of the interventions

*Similarities between the two interventions*

Both interventions were delivered in a group format and included psychoeducation about ADHD and closely related difficulties (e.g., difficulties with planning, organising, structuring daily routines and stress). Challenges and strengths with ADHD were discussed. PowerPoint presentations were used, group discussions were included, and the participants received homework assignments between the sessions.

Structured skills training group

| **Session** | **Themes and content** |
| --- | --- |
| 1 | *Introduction:* Information about the treatment and psychoeducation about ADHD. |
| 2 | *Neurobiology* *and mindfulness I:* Neurobiology of ADHD and introduction to mindfulness. Mindfulness training is included as part of all later sessions. |
| 3 | *Homework and mindfulness II:* Rationale for the use of homework in the treatment. Strategies for accomplishing home assignments are discussed. |
| 4 | *Acceptance and mindfulness III*: Acceptance is introduced and practiced. |
| 5 | *Chaos and control:* Discussion about difficulties in organization and planning. Strategies for how to manage these difficulties are introduced and practiced. |
| 6 | *Emotions:* Learning about emotions, including practice in identifying, observing and describing emotional signals in order to better manage emotions. |
| 7–8 | *Behavioural analysis:* Introduction to behavioural analysis. Strategies to find alternative behaviours are discussed, practiced and applied to own examples. Behavioural analysis is thereafter used throughout the treatment. |
| 9 | *Medication, mental illness and how to increase wellbeing:* Information about pharmacological treatment for ADHD. Symptoms of depression and other emotional problems are discussed. Information and discussions about treatment options and preventive strategies. |
| 10 | *Impulsivity, risk behaviours and addiction:* Symptoms of addiction and other forms of risk behaviours are discussed. Practice in identifying, describing and regulating impulsive behaviours. |
| 11 | *Stress:* Physiological reactions to stress and the relation between stress and performance are presented. Practice in identifying and learning about personal stress reactions and strategies for stress management. |
| 12–13 | *Self-esteem and relationships:* Differences between self-esteem, self-confidence and self-respect are clarified, including the impact of ADHD on these areas. Social skills are taught and practiced. |
| 14 | *Retrospect and outlook:* The participants summarize their experience of the group treatment, evaluate their own progress and plan for how to continue their work outside the treatment. |

| **Session** | **Themes and content** |
| --- | --- |
| 1 | *What is ADHD?* Psychoeducation about ADHD including etiology, neurobiology, symptomatology, difficulties and strengths with ADHD and examples of famous people with the diagnosis. |
| 2 | *Take charge over your daily life:* Information about how to structure daily life routines and the importance of sleep, food and activity. Stress management and problem-solving skills are presented. |
| 3 | *Take charge over your ADHD:* Deepened and further work with the problem-solving model, following the structure: Stop and think, get organized, use tools (e.g., mobile apps), use support and coaching. |

Psychoeducational control intervention

*ADHD* Attention deficit/hyperactivity disorder.
