# Supplementary material for: Evaluation of a structured skills training group for adolescents with attention-deficit/hyperactivity disorder: a randomised controlled trial
Source: Eur Child Adolesc Psychiatry. 2021 Mar 15;31(7):1–13. doi: 10.1007/s00787-021-01753-2 (PMC9343260; doi:10.1007/s00787-021-01753-2)
Supplement: Supplementary file 4 — Electronic supplementary material 4 (DOCX 14 kb) [file 787_2021_1753_MOESM4_ESM.docx]

**Supplement S4** Adherence ratings

The SSTG therapists were instructed to videotape the group sessions to enable assessment of manual adherence. Due to technical problems, four out of thirteen groups did not have their sessions recorded ^a^. Adherence ratings were performed on three sessions from each of the remaining nine groups (27 sessions in total). The rated sessions were selected on the basis that they included focus on different DBT-elements (such as mindfulness, acceptance, behavioural analysis and social skills) to ensure that all SSTG delivered these core elements of the treatment. Accordingly, sessions 3 (mindfulness), 4 (acceptance) and 7 (behavioural analysis) were selected in the first hand. However, if recordings of one of these sessions were missing, they could be replaced by sessions 2 (mindfulness), 8 (behavioural analysis), 10 (behavioural analysis in connection to impulsivity), or 12 and 13 (social skills).

Adherence was rated on a five-point scale, where 1 = unacceptable, 2 = insufficient, 3 = acceptable, 4 = good, and 5 = excellent. Two clinical psychologists with expertise in CBT and the SSTG method performed the adherence ratings. First, three sessions were inter-rated, with subsequent discussions to reach agreement on how each item should be assessed. The remaining sessions were then divided between the two psychologists and rated individually. The average adherence to the manual was considered to be acceptable to good in all sites

(*M* = 3.57, *SD* = 0.34)

^a^ No significant outcome differences were found between the groups that did not record their sessions and those that did. Therefore, all groups were included in the analyses.
